# Follow-Up Biomarkers in the Evolution of Prostate Cancer, Levels of *S100A4* as a Detector in Plasma

**DOI:** 10.3390/ijms24010547

**Published:** 2022-12-29

**Authors:** Maria Jesus Alvarez-Cubero, Elena Arance, Esperanza de Santiago, Pilar Sanchez, Maria Rosario Sepúlveda, Raquel Marrero, Jose Antonio Lorente, Jose Maria Gonzalez-Cabezuelo, Sergio Cuenca-Lopez, Jose Manuel Cozar, Fernando Vazquez-Alonso, Luis Javier Martinez-Gonzalez

**Affiliations:** 1 GENYO, Centre for Genomics and Oncological Research: Pfizer, University of Granada, Andalusian Regional Government, Genomics Unit, PTS Granada, Avenida de la Ilustración 114, 18016 Granada, Spain; 2 Nutrition, Diet and Risk Assessment Group, Bio-Health Research Institute (Instituto de Investigación Biosanitaria ibs.GRANADA), Avenida de las Fuerzas Armadas 2, 18014 Granada, Spain; 3 Department of Biochemistry and Molecular Biology III, Faculty of Medicine, University of Granada, PTS Granada, 18016 Granada, Spain; 4 Department of Cell Biology, Faculty of Sciences, University of Granada, Avenida de la Fuente Nueva S/N CP, 18071 Granada, Spain; 5 Department of Legal Medicine and Toxicology, Faculty of Medicine, University of Granada, PTS Granada, 18016 Granada, Spain; 6 Meridiem Seeds, Research and Development Department, 04710 Almería, Spain; 7 Urology Department, Virgen de las Nieves Hospital, 18014 Granada, Spain

**Keywords:** aggressiveness, biomarker, liquid biopsy, prostate cancer

## Abstract

The management and screening of prostate cancer (PC) is still the main problem in clinical practice. In this study, we investigated the role of aggressiveness genetic markers for PC stratification. We analyzed 201 plasma samples from PC patients and controls by digital PCR. For selection and validation, 26 formalin-fixed paraffin-embedded tissues, 12 fresh tissues, and 24 plasma samples were characterized by RNA-Seq, immunochemistry, immunofluorescence, Western blot, and extracellular-vesicles analyses. We identified three novel non-invasive biomarkers; all with an increased expression pattern in patients (*PCA3*: *p* = 0.002, *S100A4*: *p* ≤ 0.0001 and *MRC2*: *p* = 0.005). *S100A4* presents the most informative AUC (area under the curve) (0.735). Combination of *S100A4*, *MRC2*, and *PCA3* increases the discriminatory power between patients and controls and between different more and less aggressive stages (AUC = 0.761, *p* ≤ 0.0001). However, although a sensitivity of 97.47% in *PCA3* and a specificity of 90.32% in *S100A4* was reached, the detection signal level could be variable in some analyses owing to tumor heterogeneity. This is the first time that the role of S100A4 and *MRC2* has been described in PC aggressiveness. Moreover, the combination of *S100A4*, *MRC2*, and *PCA3* has never been described as a non-invasive biomarker for PC screening and aggressiveness.

## 1. Introduction

Prostate cancer (PC) accounts for 43% of all cancer cases in men, which amounts to more than one in five in new diagnoses. However, survival rates (98%) are the highest compared with other tumors [1]. There is controversial data about the different rates among countries suggesting that prostate-specific antigen (PSA) tests could be one of these reasons [2]. PSA shows some disadvantages; a randomized controlled trial in the USA demonstrated PSA-testing decreasing PC deaths [3]. However, PC is detected on repeated biopsy in 10–35% of patients with a negative first biopsy. Widespread PSA testing also leads to the diagnosis of clinically insignificant tumors, resulting in potential overdiagnosis and overtreatment [4].

There is a great clinical necessity for an accurate detection of PC, not only in screening stages reducing unnecessary biopsies, but also in PC management and monitoring. Therefore, new PC biomarkers have been described in recent years. Emerging biomarkers include those applying serum, urinary, or tissue samples as a test substrate [5], such as *PCA3* and *TMPRSS2-ERG* gene fusions, which are specific biomarkers that are measurable in urine. They are usually found overexpressed in PC cells in comparison to normal or benign prostate cells [6,7]. *PCA3* has been suggested in many studies as one of the most promising non-invasive methods with acceptable sensitivity and specificity in PC diagnosis, distinguishing between patients and healthy individuals [8]. It has been indicated as one of the candidates to be established as a non-invasive biomarker in diagnosis and prediction of clinical PC outcomes [9].

Other markers, such as *MRC2* (also known as *uPARAP*, *Endo180*, or *CD280*), are involved in extracellular matrix remodeling by mediating collagen degradation, and have many roles in different tumors or diseases. Data are reporting that high expression of *MRC2* produces tumor growth and is related to metastasis; thus, it usually associated with worse prognosis in several cancers [10]. Similar happens with *S100A4*, whose expression has been related as the most significant predictor of patient survival and a possible effective disease progression marker. Accumulating research evidence indicates overexpression patterns correlated with patient outcomes in gastric, colorectal, and renal cells, among others. Moreover, it has also been associated with patient survival in other tumor types, such as ovarian, pancreatic, bladder, and hepatocellular, among others [11]. Recently, the role of *S100A4* has been suggested to be a key element in the regulation of bone metastasis [12]. Moreover, Shiqin L. et al. indicated that *CASP8* and *S100A4* are protein candidate biomarkers for high-risk PC, although this study ultimately only found significant values in *CASP8* [13]. There are currently no conclusive data on both markers in PC, with this article being the first case in which a strong effect of *MRC2* and *S100A4* on PC aggressiveness is reported.

Recent massive strategies provide large amounts of data of novel biomarkers, such as *PTGFR, NREP, SCARNA22, DOCK9, FLVCR2, IK2F3, USP13*, and *CLASP1,* as potential biomarkers to predict PC progression [14], or *YWHAZ* in PC aggressiveness [15]. Moreover, machine learning models also reported new biomarkers related to PC development and progression such as *PIAS3* and *UBE2V2* [16]. However, none of them are currently used in clinical routine. 

One of the main aims of current medicine is to avoid invasive methods; that is why a liquid biopsy is increasing as one of the most universal methodologies for monitoring and stratifying tumors such as PC. Liquid biopsy is one of the most efficient non-invasive modalities which can be performed and monitored in real-time, useful for early tumor diagnosis, therapeutic guidance, and recurrence monitoring [17]. Many molecules can be analyzed in liquid biopsy like circulating tumor cells, microvesicles, circulating cell-free DNA, circulating cell-free RNA, or miRNAs [18]. Friedemann et cols. indicated that *RASSF1A* and *GSTP1* methylation patterns in circulating cell-free DNA from serum offer superior sensitivity of epigenetic biomarker analyses in the early detection of PC metastases [19]. Furthermore, genomic alterations of *RB1*, *TP53*, *MYC*, and DNA repair pathways are detected in liquid biopsy and associated with poor clinical outcomes in PC patients [20]. However, RNA levels are also a good strategy for non-invasive monitoring, enabling the determination of prognostic individualized therapy for aggressive or progressive PC [21]. Furthermore, there are commercial tests like AdnaTest, which allowed the analysis of the androgen receptor mRNA, which helps predict patients’ survival [22].

Here, we reinforce the role of *PCA3* as a non-invasive biomarker for PC and indicate for the first time the role of two other genes, *MRC2* and *S100A4*, as good aggressiveness biomarkers in PC. Moreover, the combination of these three genes improves their accuracy as liquid biopsy biomarkers for PC aggressiveness. Tumor biomarkers play important roles in tumor growth, invasion, and metastasis, and genomic ones are crucial, being one of the most informative non-invasive strategies. Present data could reinforce the role of genetic markers in liquid biopsy to improve the current aggressiveness classification. The strategy of using genetic non-invasive biomarkers for PC management could improve precision medicine among all these patients. 

## 2. Results

**Figure 1:**
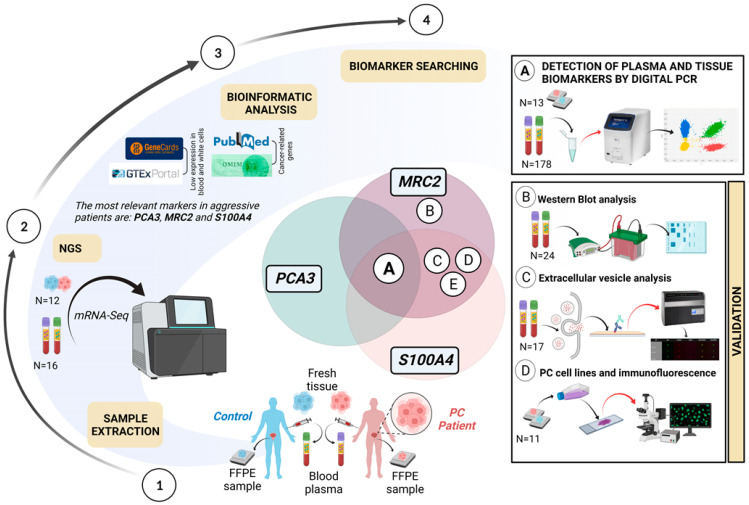
Design and methodological patterns of the present study. It shows how several methodologies and determinations were conducted to validate the results. (A) digital PCR analysis in tumor tissues and confirmed by a pathologist in healthy tissue; (B) *MRC2* proteins analysis in plasma; (C) microvesicles selection and dyeing in *MRC2* and *S100A4* for visualizing in cytometry; (D) immunofluorescence in cell lines and tissues for *MRC2* and *S100A4*; (E) immunohistochemistry in PC tissue (*MRC2* and *S100A4*). Previously, markers were selected by NGS analysis (2) by using filters obtained from different resources.

A summary representation of the present methodological study is illustrated in Figure 1. As can be seen, the present article was carried out in two relevant stages.

### 2.1. Phase 1: Biomarker Searching

#### 2.1.1. RNA Analysis and Gene Prioritization

RNA-Seq analysis provided about 500 genes. Those genes with higher expression patterns compared to healthy tissues (n = 6) were selected. At least 250 genes were chosen in each patient, always with an adjusted *p*-value below 0.05 and log2FoldChange out of range −0.10/0.10. 

Afterward, only those present in at least two plasma samples of PC patients were picked, reducing the total to 207 genes. Subsequently, using the GeneAnalytics web tool from GeneCards, genes were categorized by disease, and those included in cancer and PC categories were selected, reducing the list to 27 genes.

Finally, using related published articles, those genes previously described in plasma and cancer were selected for analysis (details in Supplementary Table S2). According to function, pathway, and previous studies from the gene set, we solely focused the analysis on *S100A4*, *MRC2*, and *PCA3*.

#### 2.1.2. Differential Gene Expression in CP Patients Versus Controls

A total of 178 plasma samples (n = 89 patients and n = 89 controls) were analyzed for gene expression by dPCR. All three candidate biomarker genes (*PCA3*: *p* = 0.002, *S100A4*: *p* ≤ 0.0001, and *MRC2*: *p* = 0.005) demonstrated statistically significant differences in expression between PC patients and healthy controls in plasma samples (see details in Supplementary Figure S1).

Among patients, we have also performed comparisons between ISUP grade (abbreviated ISUP hereinafter) (1–2 vs. 3–5), PSA (<20 vs. >20 ng/mL), and the presence of metastasis. According to these groups, we have found that *S100A4* is the best biomarker with statistically significant data when comparing ISUP (*p* = 0.044), PSA (*p* = 0.017) and metastasis (*p* = 0.025), whereas *PCA3* is only statistically significant in PSA (*p* = 0.008) and *MRC2* in none of the previous comparisons. Moreover, we have also developed the combination of all these genetic markers with clinical parameters, and those combined with *S100A4* reached statistical significance (Table 1).

Subsequently, we selected formalin-fixed paraffin-embedded (FFPE) tissue samples of patients and controls to validate that these patterns are followed in PC tissue-affected areas. These samples were marked by a pathologist indicating tumor and non-tumor areas; both areas were selected for the analysis to correctly compare expression patterns. As can be seen in Supplementary Figure S2, *S100A4* is the one with the highest expression patterns in patients’ samples.

### 2.2. Phase 2: Validation of the Biomarkers

#### 2.2.1. Western blot Analysis

It also demonstrated, as did previous data in genomic analysis, that blood samples of severe patients have a higher intensity of the band in MRC2 compared to moderate and controls (Supplementary Figure S3), as well as more expression patterns as can be seen in Supplementary Figure S4, *p* = 0.002.

#### 2.2.2. Extracellular Vesicles Analysis

**Figure 2:**
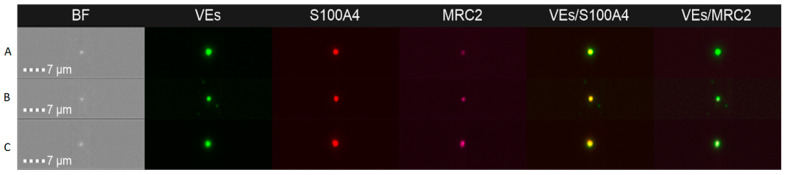
Image gallery of extracellular vesicles (EVs) from blood plasma of patients acquired by imaging flow cytometer. Brightfield (BF) images, Bodipy-labeled EVs (green), S100A4-AF594 (red), MRC2-Cy5 (magenta), and images corresponding to superimposed fluorescence channels (merged images) are shown. (**A**) Control; (**B**) moderate PC (ISUP ≤ 3); and (**C**) aggressive PC (ISUP > 3).

Lastly, we analyzed positive *S100A4* and *MRC2* extracellular vesicles (EVs) extracted of plasma from patients and healthy donors (n = 23). Due to the difficulty of finding EVs in plasma, we reinforced this point using three strategies: (I) use of ImageStream MKII (Amnis, Luminex) (Figure 2); (II) analysis of the data with IDEAS^®^ software v.6.0 (selecting easily those EVs with double events); (III) estimate of the number of objects/mL for an exact calculation of EVs (Supplementary Figures S5 and S6).

As can be seen, plasma EVs are higher in PC samples than in controls, having double the positive markers in 60.000 acquired events (Figure 2). Similarly, it was proven with similar patterns in renal cancer in our group [23,24] (details in Supplementary Figures S5 and S6). The same pattern is proven with ImageStream and IDEAS^®^ v.6.0; *S100A4* fluorescence is increased in patients versus controls, not significant values. In *MRC2*, this pattern is repeated with significant values (*p* = 0.0356). However, we should be cautious due to the small sample size in these analyses.

#### 2.2.3. Immunohistochemistry and Immunofluorescence Analysis

Firstly, we evaluated the ability of *S100A4* and *MRC2* as biomarkers by analyzing tumor and healthy prostatic regions with immunohistochemical techniques (n = 11). We discovered some differences in the staining of *MRC2*, and a Western blot was performed in this marker to develop a non-invasive quantification. Then, *MRC2* and *S100A4* gene expression analysis was undertaken using tumor and healthy paraffin-embedded prostatic tissue (n = 13). In this case, we determined a significant difference for *S100A4* and *MRC2*, detecting an increased expression of both molecules for the tumor group, but conclusive results could only be obtained for *MRC2* (details in Supplementary Figures S7–S9).

#### 2.2.4. Sensitivity and Specificity and Biomarker Correlation with Overall Survival or Progression-Free Survival

**Figure 3:**
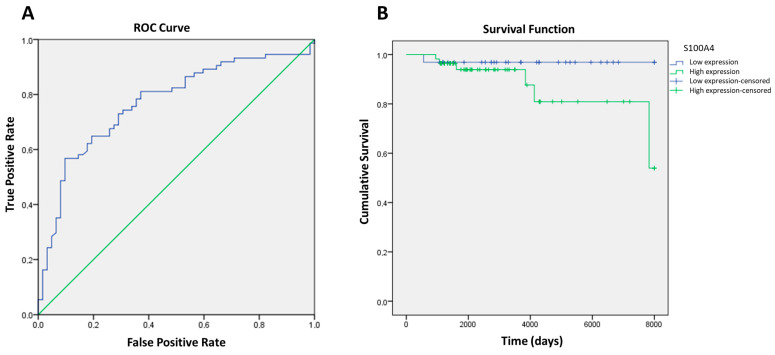
Characteristics of *S100A4* as a biomarker in plasma. (**A**) ROC (Receiver operating characteristic) curves analysis in *S100A4*; (**B**) overall survival in days calculated to *S100A4* optimal cut-point.

Given the high expression of *PCA3*, *MRC2*, and *S100A4*, and according to all overall results, we have performed a ROC curve analysis and calculated the AUC to investigate their individual ability to discriminate between PC patients and controls (see Figure 3). We also combined these genes to examine their potential diagnostic advantages. An AUC with a 95% of confidence interval (CI) was obtained to evaluate diagnostic accuracy of the individual blood-derived circulating mRNAs of these genes. In our study population overall survival (OS) rates were 96.6% at 3 years, 95.1% at 10 years, and 88.6% at 20 years. *S100A4* was significant for OS, showing reasonable specificity and sensitivity values to predict disease progression.

Although all markers are statistically significant (see Table 1) *S100A4* is the biomarker with the best performance, with an AUC value of 0.735 (95% CI = 0.668–0.803) and a specificity of 90.32%. Analysis of the other genes did not show a high discriminatory capacity (AUC < 0.70). However, the different combinations of present genes analyzed in blood provide slightly additional benefits with AUC levels > 0.74 (details in Table 2).

## 3. Discussion

Fluids biomarkers currently in use for diagnosis, prognosis, and relapse-monitoring of localized PC remain centered around PSA, with new strategies for genomic biomarkers still presenting a challenge [24]. At present, the established prognostic factors (ISUP, stage, and PSA) are insufficient to separate PC patients with high risk of cancer progression; moreover, molecular markers for metastatic propensity remain elusive [25]. RNA biomarkers are experiencing an increased interest in predicting progression-free survival and OS [26]. In the present article, we reinforced the role of *PCA3*, *MRC2*, and *S100A4* in combination, and *S100A4* on its own, as RNA biomarkers in PC aggressiveness stratification.

In PC, the role of *S100A4*-antibody therapy and its clinical applicability in treating immunosuppressive PC patients has previously been suggested [25]. Its role in promoting cell proliferation and epithelial–mesenchymal transition characteristics in tumor cells, inducing regulating bone metastasis, has also been proposed [12]. However, there are no reports on its predictive role as an aggressiveness non-invasive biomarker; as we have proven in present article, it has an AUC value of 0.735 (95% CI = 0.668–0.803) and a specificity of 90.32%. Moreover, there are no reports of its increased efficiency at screening the non-invasive biomarkers combination of *PCA3*, *MRC2*, and *S100A4,* with an AUC value of 0.761 (95% CI = 0.688–0.835) and specificity of 95.65%.

Although previous studies reported that type I transmembrane collagen receptor *Endo180* (*CD280*, *CLEC13E*, *KIAA0709*, *MRC2*, *TEM9* or *uPARAP*) was a strong prognostic indicator for PC survival [27], this is the first time that its role in PC aggressiveness is proved in blood. The role of *PCA3* is known, however is not currently used in large-scale clinical practice [28]. We employed dPCR to measure *PCA3*, *MRC2*, and *S100A4* expression in different grades of PC patients. Our results demonstrate the value of these genes as PC aggressiveness biomarkers. Moreover, Western blot analysis reveals the same expression patterns described in dPCR. The strength of this project is the consistency of data obtained from FFPE, fresh tissues, and blood samples analysis. Furthermore, concerning exosome studies from blood samples, *S100A4* allows us to detect the most aggressive stages with high specificity (90.32, *p* ≤ 0.0001) as previously reported in blood-derived non-extracellular vesicles.

One of the most critical challenges of current medicine is to offer more individualized and personalized medicine to each patient. Several biomarker-based screening kits, such as 4Kscore^®^ Test (blood) and Select MDx (urine), focus on reducing unnecessary biopsies as well as predicting the risk of developing PC in combination with clinical data. None of these screening biomarkers are used in routine clinical practice. Although precision medicine has come a long way to ameliorate cancer disease management, screening strategies in PC patients are still limited. This study validates the potential of *PCA3*, *MRC2*, and *S100A4* biomarkers in the screening of PC aggressive stages as an easy, efficient, and not high-cost method, using sensitive dPCR assays in blood samples.

Limitations of our study include the sample size in some experiments; nevertheless, a high number of different methodological analyses approve of the same hypothesis and follow the same pattern. We highlight that, firstly, the analysis was conducted with a massive strategy (RNA-Seq); its costs and the vast data of its analysis assure the success of the following steps. Subsequently, those analyses developed with smaller sample numbers performed in FFPE samples (such as immunohistochemistry/fluorescence), were conducted just as a proof of concept of the previous analysis.

## 4. Materials and Methods

### 4.1. Patients and Sample Collection

A total of 89 PC plasma samples, selected from an extended collection of samples of PC 312 (C.0005252), were used for molecular analysis. We have recruited clinical data from all patients such as T-stage, serum PSA levels, ISUP, and D’Amico risk (low, intermediate, and high risk) (details in Supplementary Table S1). Only patients with PSA ≥ 4.0 ng/mL who met the criteria for undergoing a prostate biopsy were recruited from 2012 to 2014 and included in this study; those with negative biopsy results were included as controls. All patients had localized diseases and treatment-naive at the time of the collection. All individuals underwent a systematic 20-core ultrasound-guided biopsy to limit the false negative rate. Moreover, we have collected buccal swabs and fresh tissue samples. All samples (blood, fresh tissues, and buccal swabs) were stored at −80 °C until they were processed. FFPE tissue samples were also collected from 13 samples (n = 5 controls vs. 8 patients) according to their clinical aggressiveness (n = 4, ISUP > 3; and n = 4, ISUP ≤ 3). Eighty-nine plasma samples of control individuals (mean age of 66) were also analyzed. For more details on sample analysis and selection see Figure 1. The study was approved by the Research Ethics Committee of Granada Center (CEI-Granada internal code 1503-M2-20) following the Helsinki ethical declaration and all study participants provided written informed consent before being enrolled.

### 4.2. Methodology

As was previously described in the results section, we divided the methodology into the same two stages. Each section has a specific protocol and methodology design; the second phase (validation), especially, is performed in fewer samples just to validate the data obtained in the first part of the biomarkers search.

#### 4.2.1. Biomarker Searching

This section includes: (I) RNA extraction and quantification according to the QIAamp RNA miRNeasy Serum/Plasma protocol (QIAGEN, Hilden, Germany); (II) NGS analysis using TruSeq Stranded mRNA Library Prep kit (Catalog # 20020594) (Illumina, San Diego, CA, USA); (III) bioinformatic analysis and potential biomarkers searching using the reference genome GRCH38 by STAR tool (see all details in Supplementary Material). Moreover, in silico analysis validation using Kyoto Encyclopedia of Genes and Genomes (KEGG), Gene Ontology (GO), PathCards Pathway UD, and Reactome is detailed in Supplementary Table S3. 

The main strategy of detection is focused on digital PCR (dPCR) analysis in plasma and tissue samples by a QuantStudio ™ 3D Digital PCR System (Waltham, MA, USA). Details of probes in Supplementary Table S4.

#### 4.2.2. Validation of the Biomarkers

This section includes: (I) Western blot; (II) EVs analysis; (III) immunohistochemistry and immunofluorescence in cell lines and samples analysis. All these techniques were carried out following conventional procedures (all details in Supplementary Material). Moreover, just to remind, several methodologies such as Western blot are limited to those markers that produce proteins or validate immunohistochemistry analysis (details in Supplementary Table S3).

## 5. Conclusions

The present study indicates for the first time the use of *PCA3*, *MRC2*, and *S100A4* RNA biomarkers in blood for PC aggressiveness. Moreover, *S100A4* on its own could serve as an accurate biomarker in PC management of high-grade patients screening. These biomarkers, in combination with clinical data, could offer promising strategies to anticipate the follow-up of aggressive patients. Current clinical strategies are lacking in the application of non-invasive molecular biomarkers in PC patients’ stratification. The present discovery opens a new approach in non-invasive genetic monitoring of PC, suggesting the inclusion of these genes as markers in clinical routine.

## Figures and Tables

**Table 1:** Clinical statistical analysis of present markers.

	Clinical Values	n (0)	n (1)	AUC	95% CI	PPV	NPV	Sensitivity	Specificity	*p*
*MRC2 & S100A4*	ISUP grade	20	31	0.648	0.491–0.804	74.07%	54.16%	64.52%	65.00%	**0.039**
	PSA	26	25	0.6	0.443–0.557	100.00%	56.52%	20.00%	100.00%	**0.016**
	Metastasis	12	34	0.669	0.494–0.845	86.95%	39.13%	58.82%	75.00%	**0.044**
*PCA3 & S100A4*	ISUP	22	37	0.561	0.410–0.713	69.23%	42.42%	48.65%	63.64%	0.358
	PSA	27	29	0.601	0.452–0.750	80.00%	54.33%	27.58%	92.59%	**0.049**
	Metastasis	16	38	0.681	0.519–0.842	82.35%	50.00%	73.68%	62.50%	**0.012**

n (0) corresponds to ISUP (1–2), PSA (<20), and no metastasis; n (1) corresponds to ISUP (3–5), PSA (>20), and presence of metastasis; AUC (area under the curve); CI (confidence interval); PPV (positive predictive values); NPV (negative predictive values).

**Table 2:** ROC parameters for diagnosis of PC patients in serum biomarkers alone and combined.

Biomarker	AUC	95% CI	PPV (%)	NPV (%)	Sensitivity (%)	Specificity (%)
*MRC2*	0.622	0.574–0.670	59.26	100	100	24.36
*PCA3*	0.644	0.584–0.703	63.63	90.90	97.47	31.25
*S100A4*	*0.735*	0.668–0.803	87.5	63.63	56.76	*90.32*
*MRC2-PCA3*	0.759	0.692–0.826	69.66	94.28	96.88	55.00
*MRC2-S100A4*	0.747	0.673–0.0820	89.19	66.21	56.90	92.45
*PCA3-S100A4*	0.745	0.675–0.815	92.31	60.27	55.38	93.62
*MRC2-PCA3-S100A4*	*0.761*	0.688–0.835	93.75	65.67	56.60	*95.65*

## Data Availability

Data are available for bona fide researchers who request it from the authors. The methods and procedures for diagnosis are disclosed in the patent application ES202230187, so data sharing is also limited to this patent.

## Supplementary Materials

The supporting information can be downloaded at: https://www.mdpi.com/article/10.3390/ijms24010547/s1. References [29,30,31,32,33] are cited in the supplementary materials.
