# Supplementary material for: Follow-Up Biomarkers in the Evolution of Prostate Cancer, Levels of S100A4 as a Detector in Plasma
Source: Int J Mol Sci. 2022 Dec 29;24(1):547. doi: 10.3390/ijms24010547 (PMC9820153; doi:10.3390/ijms24010547)
Supplement: Supplementary file 1 [file ijms-24-00547-s001.zip › ijms-2028740-supplementary.pdf]

Supplementary Material

SUPPLEMENTARY NOTE

Phase 1: Biomarker searching

RNA extraction and quantification

All individuals underwent a systematic 20-core ultrasound guided biopsy in order to limit the false negative rate. Two of those biopsy cylinders of each patient were taken for developing molecular analysis; they were frozen at -80ºC until their extraction. RNA was extracted using the QIAamp RNA miRNeasy Mini Kit (Qiagen/Qiacube). Moreover, we also extracted RNA from blood samples, for developing liquid biopsy validation. Firstly, blood samples were centrifuged during 5 minutes at 1400g and 4 °C for plasma separation; and stored at -80°C until RNA extraction. In this case, RNA was extracted according to the QIAamp RNA miRNeasy Serum/Plasma (Qiagen/Qiacube) protocol. In both cases RNA was quantified using a NanoDrop™ 2000c spectrophotometer (Thermo Scientific) and RNA quality were assessed with 2100 Bioanalyzer using RNA 6000 Nano Kit (Agilent Technologies Ltd).

Next Generation Analysis

Strand-specific mRNA-Seq libraries were generated with TruSeq Stranded mRNA Library Prep kit (Catalog # 20020594, Illumina) using 1µg of total RNA input, according to the manufacturer's instructions. The Poly-A containing mRNA molecules are purified using poly-T oligo attached magnetic beads. The purified mRNA was fragmented and copied into first strand cDNA, followed by second strand cDNA synthesis. In the next step an adenine (A) nucleotide to the 3' ends were added and the dual-index adapters were ligated. Finally, the products were enriched with PCR and purified. Indexed DNA libraries were quantified using a Qubit® fluorometer and Qubit® dsDNA HS Assay Kit (Invitrogen™/Thermo Fisher Scientific, Carlsbad, CA, USA) and the quality was

checked by using an Agilent 2100 Bioanalyzer and High Sensitivity DNA Kit (Agilent Technologies Ltd.). Final libraries were normalized, pooled in equal volumes, and sequenced on Illumina's NextSeq 500 platform using a paired-end read (2 x75 bp) protocol with the Illumina 150 cycle High-Output reagent kit which generated approx. 25 million reads for each library. A total 12 biopsies samples (6 controls and 6 PC) and 4 plasma samples of PC were analyzed by NGS. Clinical mean values were ISUP (3.7 (range from 5 to 1)), high risk, 8/10 have metastasis.

Bioinformatic analysis and potential biomarkers searching.

After the massive sequencing, samples were aligned using the reference genome GRCH38 by STAR tool. For the differential expression analysis, we used the R environment following DESeq method. DESeq integrates methodological advances to facilitate a quantitative analysis of comparative RNA-Seq data based on the negative binomial distribution with an adjustment for multiple tests using the Benjamini-Hochberg method. Thus, a list of genes that are significantly expressed in present analyzed PC samples was obtained (Supplementary Table S2).

Subsequently, a deep searching in databases such as GeneCards was performed to select those genes with expression patterns that are detectable among a variety of tissues such as blood and plasma cells derived from plasma. Moreover, a double check was developed in OMIM database and NCBI for confirming that selected genes are related to cancer and principally to PC.

Functional in silico analysis

In order to know the main function of these genetic markers Kyoto Encyclopedia of Genes and Genomes (KEGG) (https://www.genome.jp/kegg/ accessed on 29 November 29, 2022); Gene Ontology (GO) (http://geneontology.org/ accessed on 29 November 29, 2022); PathCards

Pathway UD (https://pathcards.genecards.org/ accessed on 29 November 29, 2022) and Reactome (https://reactome.org/ accessed on 29 November 29, 2022) were used to discover the main pathways where MRC2, PCA3 and S100A4 exercised their function (Supplementary Table S2).

Detection of plasma biomarkers by Digital PCR (dPCR)

In order to assess the efficiency of selected markers as non-invasive ones for liquid biopsy analysis, we have developed dPCR in plasma samples. cDNA was combined with PCR reagents to produce a PCR reaction in a total volume of 18 µl and using the same TaqMan® probes (see Supplementary Table 4). To assemble the chips, we follow the instructions recommended in "QuantStudio ™ 3D Digital PCR System User Guide". A 9800 dual PCR System (ThermoScientific) was used for the amplification. The PCR conditions followed manufacturer instructions. For housekeeping selection, we have developed different assays changing dilutions (1/2 and 1/10), volume (2.5 or 1 µl) and probes (*MTR* or *HPRT1*). Finally, better results were obtained with *MTR* and we have conducted the assay using this probe.

Data were analyzed using QuantStudio 3D AnalysisSuite Cloud Software. After calculating the FAM / VIC ratio of all samples and markers, a T-student statistical analysis was performed to check if there were differences between the means of the two populations, considering as significant p-value those < 0.05. Results were represented in a Box Plot graph, showing the median, the first and third quartiles and the maximum and minimum values, for each of the groups of samples (controls and cases).

Detection of FFPE biomarkers by Digital PCR (dPCR)

RNA extraction from the samples was performed following the indications of the section "Purification of Total RNA from FFPE TissueSections" of the RNeasy FFPE kit from QIAGEN®. The reverse transcription was then carried

out, to convert the extracted RNA into cDNA, using the SuperScript™ VILO™ cDNA Synthesis Kit from Thermo Fisher Scientific. In dPCR assays, specific probes of the *S100A4, MRC2* and *PCA3* genes were used (in FAM), while the endogenous *MTR* gene is marked in VIC. Data are reported as different FAM / VIC ratios, both for tumour samples and healthy controls. Chips loaded with the samples were introduced into the thermal cycler and once the PCR was performed, chips were read on QuantStudio™ 3D Digital PCR Instrument. FFPE tissue samples were marked by anatomopathologist indicating tumour and non-tumour area, both areas were selected for the analysis for comparing expression patterns correctly.

Phase 2: Validation of the biomarkers

Western blot analysis

Diluted plasma samples (1 in 30 dilution in RIPA buffer) were resuspended in 2x Laemmli buffer and heated to 95 °C for 5 min. Samples (30μL) were subjected to 4-15% sodium dodecyl sulfate-polyacrylamide gel electrophoresis (SDS-PAGE). A prestained protein molecular weight marker, (BioRad cat# 1610374), was used to calculate sample molecular weights and to monitor the progress of the electrophoretic run.

After electrophoresis, the proteins were transferred from the gel to PVDF (polyvinylidene difluoride) blotting membranes in a buffer containing 25 mM Tris, 190 mM glycine, and 20% methanol. After staining with Ponceau Red (2% Ponceau Red, 6% acetic acid), blots were saturated for 1 h at room temperature, in 5% blocking agent (dried milk in PBS-Tween 0.1%). The membrane was incubated overnight at 4 °C with the primary antibody anti-human-Endo180 (Abcam cat# ab181075, which is specific for *MRC2* gene), diluted 1:1000 in PBS-Tween 0.1% with 5 % blocking agent. Following three 5 min washes of PBS-

Tween 0.1%, the blots were incubated for 1 h with the appropriate anti Ig G-horseradish peroxidase (HRP) conjugated secondary antibody at a dilution of 1:3000 in PBS-Tween 0.1% with 5% blocking agent. After three washes in PBS-Tween 0.1%, the target proteins were visualized using Immun-Star™ WesternC™ Chemiluminescent kit (Bio-Rad). The ImageJ program (http://rsb.info.nih.gov/ij/) was used for quantitative analysis of the bands. See results of the western in Supplementary Figure S3.

It is interesting to mention that just Endo180 (*MRC2* gene) in present analysis showed relevant results in immunohistochemistry and immunofluorescence, so western blot was conducted only in this marker to analyze the protein, more details in Supplementary Table S3.

Extra vesicle (EVs) analysis

A total blood plasma volume of 800 μl per individual was used, which was centrifuged at 15,000 g at 4°C for 10 minutes. Subsequently, the supernatant was transferred to ultracentrifuge tubes (ThermoScientificSorvall X50 TubeSorvallpolyallomer 6ml) which were made up to 0.1 μm filtered PBS. We proceeded to ultracentrifuge (ThermoScientific ™ Sorvall ™ WX ULTRA SERIES centrifuge) at 160,000g at 4°C for 2 hours. The ultracentrifugation was then resuspended and repeated again. In this way, the isolation of the EVs was achieved, which were kept at -80°C until their subsequent fluorescent labeling.

Isolated EVs were incubated simultaneously with the anti-MRC2/ENDO180 (Abcam, ab70132; 1:50 PBS dilution filtered 0.1 μm) and the anti-S100A4 (Invitrogen, MA5-31333; 1:100 dilution) primary antibodies for 1 hour at room temperature. Afterwards, EVs were incubated with the anti-rabbit Cy5 (ab6564, Abcam, 1: 1000) and the anti-mouse AlexaFluor594 (A11005, ThermoFisher, 1: 1000) secondary antibodies. The BODIPY™ FL C5-Ceramide complexed to BSA (3 μM) was used as EVs membrane marker by incubation for 30 min at room

temperature. Then, samples were run through Invitrogen™ exosome spin columns (molecular weight 3000) for efficient removal of unincorporated antibodies and dye from the labelled EVs. Finally, samples were processed in the ImageStreamX Mark II image cytometer (Amnis Corporation, Seattle, Washington, USA) with the acquisition program INSPIRE version 200.1.681.0, and their subsequent analysis with the program IDEAS, Image Data Exploration and Analysis Software version 6.2. EVs were excited with 488 nm laser line (200 mW) to detect the membrane marker BODIPY, 561 nm laser line (200 mW) for S100A4-AlexaFluor594, 642 nm laser line (150 mW) for MRC2-Cy5, 785 nm laser line (5 mW) to obtain image of complexity (SSC), and LED (65 mW) to capture brightfield (BF) image, using a 60X objective and acquiring 60,000 events per sample. Acquisition of 10,000 events of each sample was carried out together with beads of 1 μm diameter without fluorescence as a size control, which allowed selecting EVs that had a smaller diameter.

PC cell lines and immunofluorescence

LNCaP cells were cultured in RPMI medium containing 10% (v/v) fetal bovine serum (FBS, GIBCO), supplemented with 2 mM L-glutamine, 100 U/ml penicillin, 100 μg/ml streptomycin. PC-3 cells were cultured in Ham's F-12K (Kaighn's) medium containing 10% (v/v) FBS and the antibiotics. Both cell types were incubated at 37°C in a humidified atmosphere of 5% CO_2. For immunofluorescence, cells were seeded at a density of 25,000 cells onto 12 mm diameter round glass coverslips coated with 0.1 mg/ml poly-L-Lysine. Cells were fixed with 4% paraformaldehyde in PBS for 20 min, permeabilized with 0.2% (v/v) Triton X-100 in PBS and blocked with 3% bovine serum albumin (BSA) in PBS for 1 h. Subcellular localization of proteins was determined by incubation with the polyclonal anti-MRC2/ENDO180 (ab70132, Abcam) and the monoclonal anti-S100A4 (CL0240, ThermoFisherScientific) primary antibodies at 1:25 dilution in 3% BSA in PBS at room temperature. After 3 washes with

PBS, the cells were incubated for 1 h at room temperature with the corresponding secondary antibodies conjugated with AlexaFluor 594 and AlexaFluor 488 (A11005 and A11001, ThermoFisherScientific, respectively), diluted 1:1000 in PBS-3% BSA. Cell nuclei were stained with DAPI (1 μg/ml) for 2 min and coverslips were mounted in FluorSave™Reagent (Calbiochem 345789). Preparations were stored at 4°C until visualization by confocal microscopy. A Zeiss LSM 710 confocal laser scanning inverted microscope (Carl Zeiss, Jena, Germany) with Zeiss ZEN 2010 software were used for fixed LNCaP and PC3 cell lines imaging. LNCaP and PC3 cells were excited with diode laser line 405 nm (30 mW) at 6% AOTF (Acousto Optical Tunable Filter) for the detection of nuclear staining with DAPI (blue) and Argon laser line 488 nm (25 mW) at 3% AOTF for the detection of MRC2-AlexaFluor488 (green). A Zeiss Plan-Apochromat 63x/1.4 NA oil-immersion DIC (Differential Interference Contrast) M27 objective and 1.0 Airy Unit pinhole were used for sequential channel acquisition of images. Images were processed with ZEN Blue Edition (Carl Zeiss, Jena, Germany) and Image J software.

Immunohistochemistry in control and PC-human samples

Paraffin-embedded tissue of human prostate was sectioned by microtomy (8 μm thickness). Tissue sections were deparaffinized and hydrated, and subjected to proteolytic-induced epitope retrieval by treatment with proteinase K (10 μg/ml) at 37ºC for 10 min. The sections were then permeabilized by immersion in 0.05% (v/v) Triton X-100 in PBS (PBS-T) for 15 min and endogenous peroxidase activity was quenched with PBS-0.5% (v/v) H_2O_2 for 45 min. After blocking with 0.2% (w/v) gelatin, 0.25% (v/v) Triton X-100 in PBS (PBS-G-T) and 0.1M Lysine for 1 h, sections were incubated with the primary anti-MRC2 (1:300, Abcam ab70132) and anti-S100A4 (1:300, Invitrogen CL0240) antibodies diluted at 1:25 in PBS-G-T overnight at room temperature in a humidified chamber at 4°C. Sections were subsequently washed in PBS-T and incubated

with biotinylated anti-rabbit or -mouse antibodies (1:600, Sigma, B8895 and B8520, respectively), and then with ExtrAvidin-peroxidase (1:200, Sigma, E2886). The immunodetection was carried out using 0.03% (w/v) 3,3-diaminobenzidine tetrahydrochloride and 0.2% H_2O_2. Sections were dehydrated and mounted with Eukitt (Panreac) for observation under an Olympus BX43 microscope with a DP22 digital color camera.

Alternatively, immunofluorescence analysis was performed with the primary antibodies described above and the corresponding AlexaFluor488 secondary antibodies (1:1000). Cell nuclei were stained with DAPI (1 μg /ml). Due to endogenous autofluorescence of prostate tissue, sections were incubated with the Autofluorescence Eliminator Reagent (Millipore), and after that mounted in FluorSave reagent for visualization by confocal microscope. Negative controls were performed for every set of experiments by omitting the primary antibodies from the procedure.

Statistical analysis

All analyses were performed using the SPSS v.22 statistical package (IBM Corporation, United States). For expression analysis, Kolmogorov-Smirnov (Lilliefors' correction) test was used to test the normalization of the samples. This test revealed that our results did not follow a Gauss distribution, therefore a non-parametric test (U-Mann Whitney test) was performed for all variables. The level of statistical significance used was $p < 0.05$. Outliers were identified by applying the Tukey's hinges approach. To establish the cut-off point with the best predictive capacity for each biomarker as well as their combination, the Youden's index was calculated from the sensitivity and specificity values of each of the coordinates obtained in the ROC curve. Once the cut-off point for each biomarker was established, sensitivity, specificity, positive predictive values (PPV) and negative predictive values (NPV) were calculated, as well as

the area under the ROC curve (AUC). The relationship between each biomarker and clinical variables was analysed using Pearson's chi-squared test or, in cases in which the applicability conditions were not met, Fisher's exact test. Survival curves were estimated with the Kaplan–Meier method (95% confidence intervals (CI)), and the differences between subgroups were compared using the log-rank test.

Shapiro-Wilks test was performed to prove normalization of the Western-Blot analysis samples. One-way ANOVA test, Scheffe multiple comparison test, and non-parametric Kruskal-Wallis test were used to determine if there are statistically significant differences between patients with severe prostate cancer, moderate prostate cancer, and control group. The level of statistical significance used was $p < 0.05$.

SUPPLEMENTARY TABLES

Supplementary Table S1. Summary of the type and characteristic of the samples.

Characteristic	PC N (%)
**Age (years)**	
**<60**	9 (10.11%)
**60-69**	20 (22.47%)
**70-79**	37 (41,57%)
**≥80**	19 (21.34%)
**NA**	4 (4.49%)
**PSA level (ng/ml)**	
**<20**	42 (47.19%)
**≥20**	35 (39.32%)
**NA**	12 (13.48%)
**ISUP grade**	
**1**	11 (12.36%)
**2**	22 (24.72%)
**3**	7 (7.86%)
**4**	17 (19.10%)
**5**	23 (25.84%)
**NA**	9 (10.11%)
**Gleason score**	
**≤7**	40 (44.94%)
**>7**	40 (44.94%)
**NA**	9 (10.11%)
**D'Amico Risk**	
**Low**	3 (3.37%)
**Medium**	21 (23.59%)
**High**	41 (46.06%)
**NA**	24 (26.96%)
**Metastasis**	
**Yes**	51 (57.30%)
**No**	21 (23.59%)
**NA**	17 (19.10%)
**Exitus**	
**Yes**	7 (7.87%)
**No**	82 (92.13%)

Footnote ST 1: NA (not available).

Supplementary Table S2. RNA analysis and gene prioritization.

Gene	baseMean	log2 FoldChange	lfcSE	p-value	padj	Described in plasma & cancer	Function
**PCA3**	1908.59	-7.541	2.223	0.001	0.460	12	Overexpression of PCA induced downregulation of PRUNE2, leading to decreased cell proliferation.
**PTPRC**	578.12	0.135	1.032	2.70E-06	2.91E-04	15	This protein tyrosine phosphatase has been shown to be an essential regulator of T- and B-cell antigen receptor signaling.
**HBB**	1409.93	0.126	1.031	2.16E-06	2.61E-04	5	Involved in oxygen transport from the lung to the various peripheral tissues.
**ILK**	7240.57	-0.305	0.626	0.027	0.125	14	Activity of this protein is important in the epithelial to mesenchymal transition. and over-expression of this gene is implicated in tumor growth and metastasis.
**ETS1**	841.98	-0.194	0.527	0.021	0.107	4	These proteins function either as transcriptional activators or repressors of numerous genes. and are involved in stem cell development. cell senescence and death. and tumorigenesis.
**S100A4**	7174.96	-0.454	0.856	0.032	0.138	15	The protein S100-A4 is involved in the regulation of a number of cellular processes such as cell cycle progression and differentiation.
**MRC2**	3617.95	-0.199	0.530	0.036	0.148	2	May play a role during cancer progression as well as in other chronic tissue destructive diseases acting on collagen turnover.
**SCAP**	911.87	-0.518	1.002	0.033	0.141	4	SCAP protein participates in sterol metabolism by binding to sterol regulatory element binding proteins.
**STOM**	5081.35	-0.521	1.078	0.020	0.107	3	The encoded protein localizes to the cell membrane of red blood cells and other cell types. where it may regulate ion channels and transporters.
**AKT1**	6328.06	-5.261	1.917	0.003	0.045	97	The AKT protein regulate a wide variety of cellular functions including cell proliferation. survival. metabolism. and angiogenesis in both normal and malignant cells.

**CDKN1A**	1028.81	-0.577	1.365	0.016	0.094	52	Plays an important role in controlling cell cycle progression and DNA damage-induced G2 arrest.
**ZEB1**	550.57	0.139	1.033	4.89E-07	1.16E-04	17	ZEB1 is involved in control of EMT both during development and tumorigenesis.
**SPARC**	7674.86	-0.568	1.282	0.019	0.102	16	The gene product has been associated with tumor suppression but has also been correlated with metastasis based on changes to cell shape which can promote tumor cell invasion.
**MALAT1**	9376.75	-0.421	0.781	0.038	0.152	9	The encoded long non-coding RNA may act as a transcriptional regulator for numerous genes, including some genes involved in cancer metastasis and cell migration, and it is involved in cell cycle regulation.
**CSF3R**	213.99	0.130	1.031	8.81E-06	0.001	3	The protein encoded by this gene is the receptor for colony stimulating factor 3, a cytokine that controls the production, differentiation, and function of granulocytes.
**TPM3**	523.80	0.345	1.109	0.001	0.030	2	This gene encodes a member of the tropomyosin family of actin-binding proteins.
**LDB1**	3108.19	-0.316	0.638	0.029	0.133	0	Discarded by Literature
**CCNH**	153.83	0.121	1.030	1.53E-05	0.001	0	Discarded by Literature
**IFI16**	6332.46	0.147	1.035	5.13E-09	9.12E-06	0	Discarded by Literature
**JPT1**	3610.22	-0.213	0.539	0.037	0.151	0	Discarded by Literature
**SNHG5**	11230.16	-2.999	2.085	0.021	0.107	0	Discarded by Literature
**BMP2K**	714.62	-0.202	0.534	0.012	0.084	0	Discarded by Literature
**NCF2**	191.94	0.127	1.031	6.85E-06	0.001	0	Discarded by Literature
**COX6C**	6958.23	-0.668	1.847	0.025	0.119	0	Discarded by Literature
**SNHG7**	5209.68	-0.231	0.555	0.018	0.099	0	Discarded by Literature
**MEX3C**	547.80	0.123	1.030	2.39E-06	2.74E-04	0	Discarded by Literature
**TLE4**	365.11	0.129	1.031	2.66E-06	2.91E-04	0	Discarded by Literature

Footnote ST 2: lfcSE (standard error of the log2FoldChange estimate); padj (adjusted p-value).

Supplementary Table S3. Details of genetic markers analysed in present work.

	**Prostate Cancer Associated 3 (PCA3)**	**S100 Calcium Binding Protein A4 (S100A4)**	**Mannose Receptor C Type 2 (MRC2)**
**Gen Differential Expression***	Overexpressed in Prostate	Overexpressed in Cerebral Cortex and Liver	Overexpressed in Whole Blood and Artery-Aorta
**Transcript**	Long non-coding RNA	--	--
**Protein**	--	C-type mannose receptor 2	Protein S100-A4**
**Pathway**	--	AKT1-BGN-COL18A1-EFEMP2-FBLN5	AREG-BTC-MDM2-S100B-TP53
**Associated Disorders**	Prostate Cancer	Bone Cancer, Bone and Neuronal disease	Gastrointestinal related Cancer, Immunorespiratory and Skin diseases
**Role in Cancer**	This gene produces a spliced, long non-coding RNA that is highly overexpressed in most types of prostate cancer. Overexpression of PCA induced downregulation of PRUNE2, leading to decreased cell proliferation [29,30]	The encoded protein plays a role as endocytotic lectin receptor displaying calcium-dependent lectin activity. May play a role during cancer progression as well as in other chronic tissue destructive diseases acting on collagen turnover [29,31]	The protein S100-A4 is involved in the regulation of a number of cellular processes such as cell cycle progression and differentiation. As cancer biomarker, the overexpression of S100A4 is an indicator of poor prognosis and high metastatic potential [29,32]

**Gene Ontology**	**Biological Process**	None	Positive regulation of intracellular signal transduction; Cell differentiation; Positive regulation of signaling/signal transduction	Endocytosis; Vesicle-mediated transport; Establishment of localization; Transport
	**Molecular Function**	None	RAGE receptor binding; Calcium-dependent protein binding; Identical protein binding; Signaling receptor binding	Carbohydrate binding
**KEGG: Kyoto Encyclopedia of Genes and Genomes**		No Hits. Remark: PCA3 is a lncRNA related with prostate cancer (hsa 05215)	Phagosome (hsa 04145)	No Hits
**PathCards Pathway UD**		None	Integrin family cell surface interactions	Ca, cAMP and Lipid Signaling
**Reactome Pathways**		No results	Cross-presentation of soluble exogenous antigens (endosomes)	Vitamin D receptor pathway

Footnote ST 3**.** *Based on mRNA differential expression (GTEx), protein differential expression (HIPED) and enrichment analysis with GO, KEGG, PathCards Pathway UD and Reactome on-line tools.

Supplementary Table S4. TaqMan® probes used for dPCR.

Gene	Probe Details
MTR	Hs01090026_m1
S100A4	Hs00243202_m1
MRC2	Hs00195862_m1
PCA3	Hs03462121_m1

SUPPLEMENTARY FIGURES

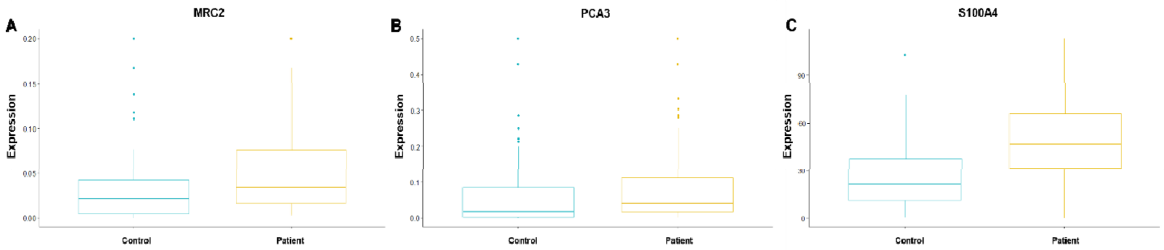

Supplementary Figure S1. Differential gene expression by dPCR in controls vs patients. A. Representation for *MRC2*; B. Representation for *PCA3*; and C. Representation for *S100A4*.

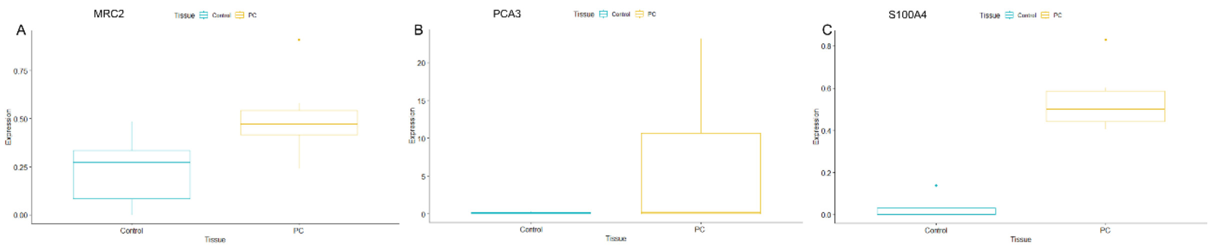

Supplementary Figure S2. Relative bar chat expression patterns in genes *S100A4* (A), *MRC2* (B) and *PCA3* (C) genes in controls (non-tumour FFPE area) and patients (tumour FFPE area). U Mann-Whitney statistical tests were performed for A and C; non-paired T-student test for B.

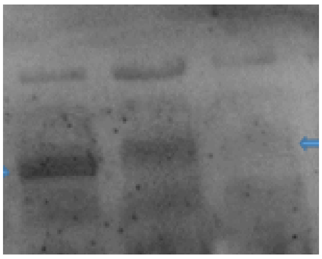

Supplementary Figure S3. Western blot results. From left to right we can see rails from severe patients (rail 1), moderate patients (rail 2) and control sample (rail 3). Plasma from the same control individuals was used as a reference housekeeping (relative *MRC2* level = 1.0) and the normalization of densitometric measurements on all analytical gels in according with data published by Palmier, *et al* [33].

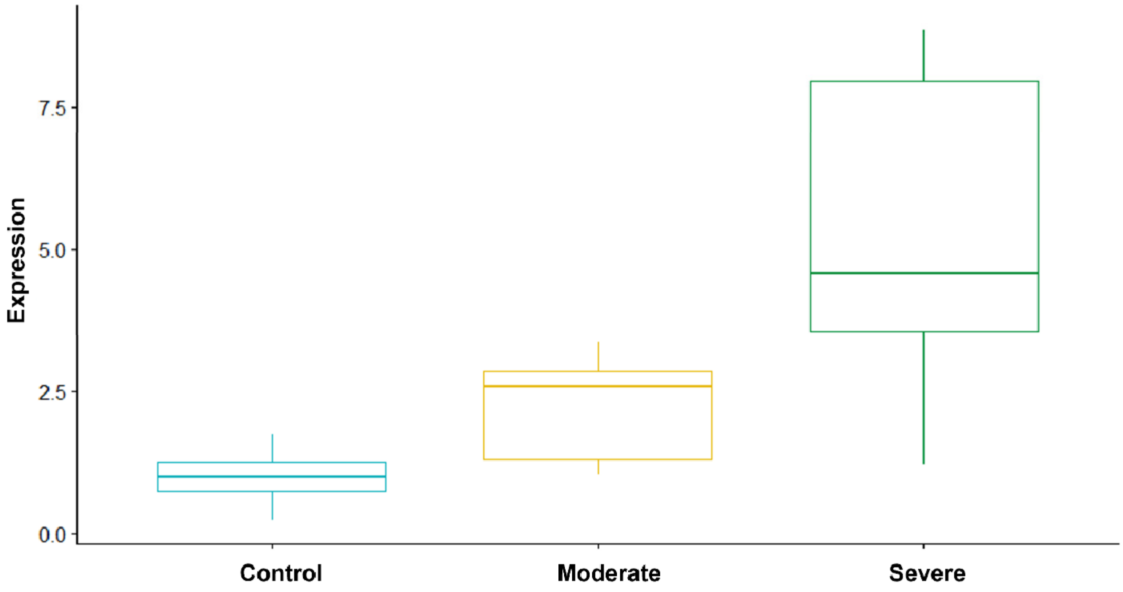

Supplementary Figure S4. Western blot analysis in blood samples represented by box plots.

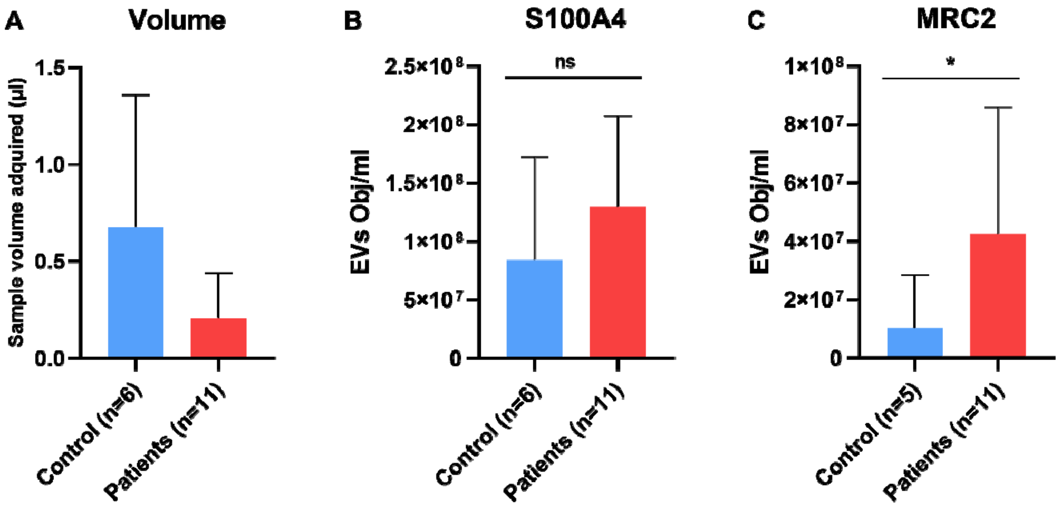

Supplementary Figure S5. Bar graphs representing the volume acquired by imaging flow cytometer (A) protein levels of *S100A4* (B) and *MRC2* (C) in EVs from plasma of patients. A represents the average volume acquired in healthy controls and PC patients. B and C represent the average objects/ml double positive to EVs/*S100A4* and EVs/*MRC2*. The statistical significance was evaluated using Mann-Whitney test for B and C. Data represent mean ± SEM. (*) $p < 0.1$; (ns) not significant.

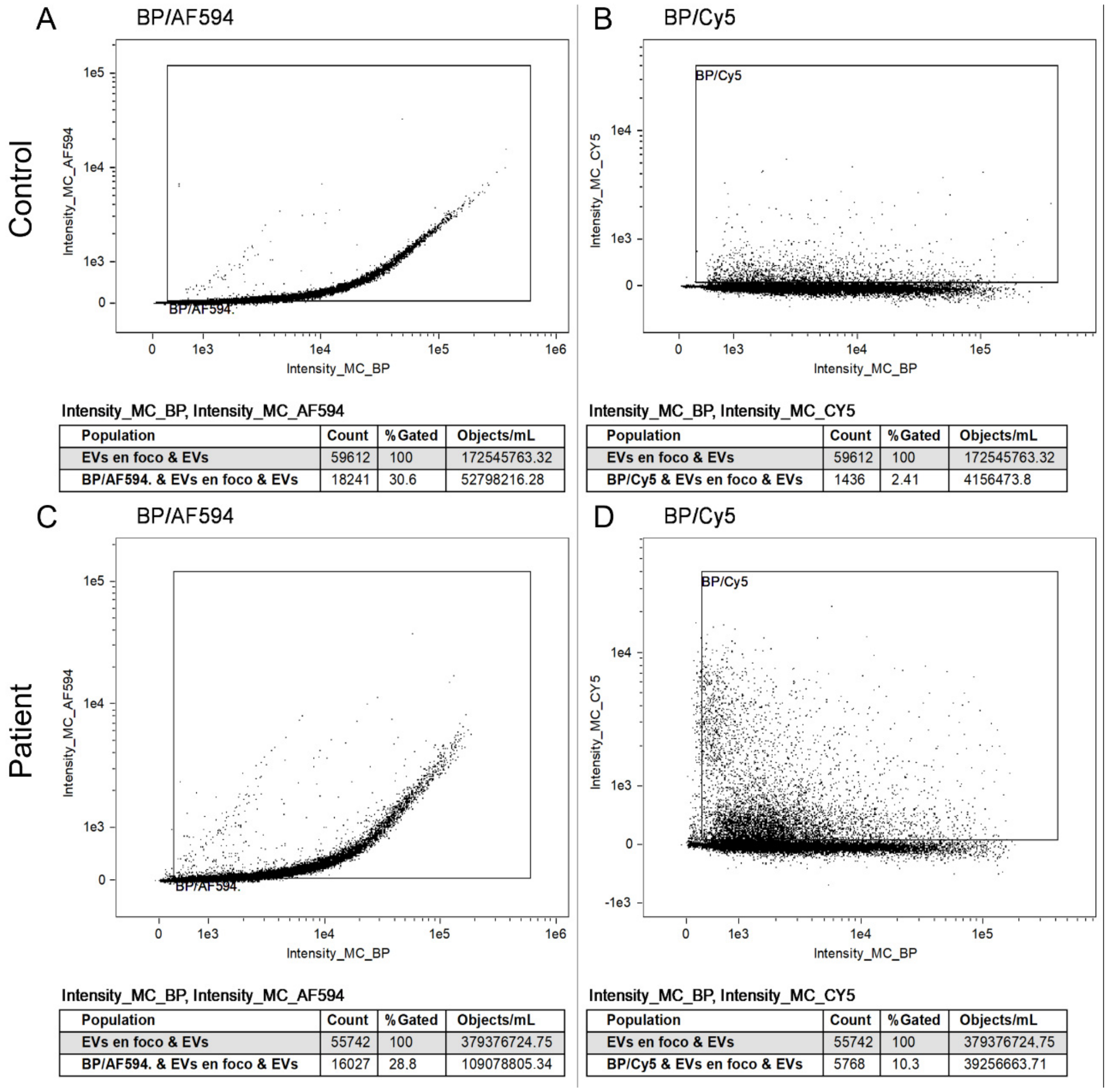

A — Intensity_MC_BP, Intensity_MC_AF594

Population	Count	%Gated	Objects/mL
EVs en foco & EVs	59612	100	172545763.32
BP/AF594. & EVs en foco & EVs	18241	30.6	52798216.28

B — Intensity_MC_BP, Intensity_MC_CY5

Population	Count	%Gated	Objects/mL
EVs en foco & EVs	59612	100	172545763.32
BP/Cy5 & EVs en foco & EVs	1436	2.41	4156473.8

C — Intensity_MC_BP, Intensity_MC_AF594

Population	Count	%Gated	Objects/mL
EVs en foco & EVs	55742	100	379376724.75
BP/AF594. & EVs en foco & EVs	16027	28.8	109078805.34

D — Intensity_MC_BP, Intensity_MC_CY5

Population	Count	%Gated	Objects/mL
EVs en foco & EVs	55742	100	379376724.75
BP/Cy5 & EVs en foco & EVs	5768	10.3	39256663.71

Supplementary Figure S6. Dot plots show the fluorescence intensity of S100A4-AF594 on the Y axis in the left column and MRC2-Cy5 on the Y axis in the right column, versus the intensity of Bodipy-labeled EVs on the X axis. Selected regions indicate the double positive events EVs/*S100A4* and EVs/*MRC2*. The samples correspond to blood plasma from a healthy person (control) and a PC patient.

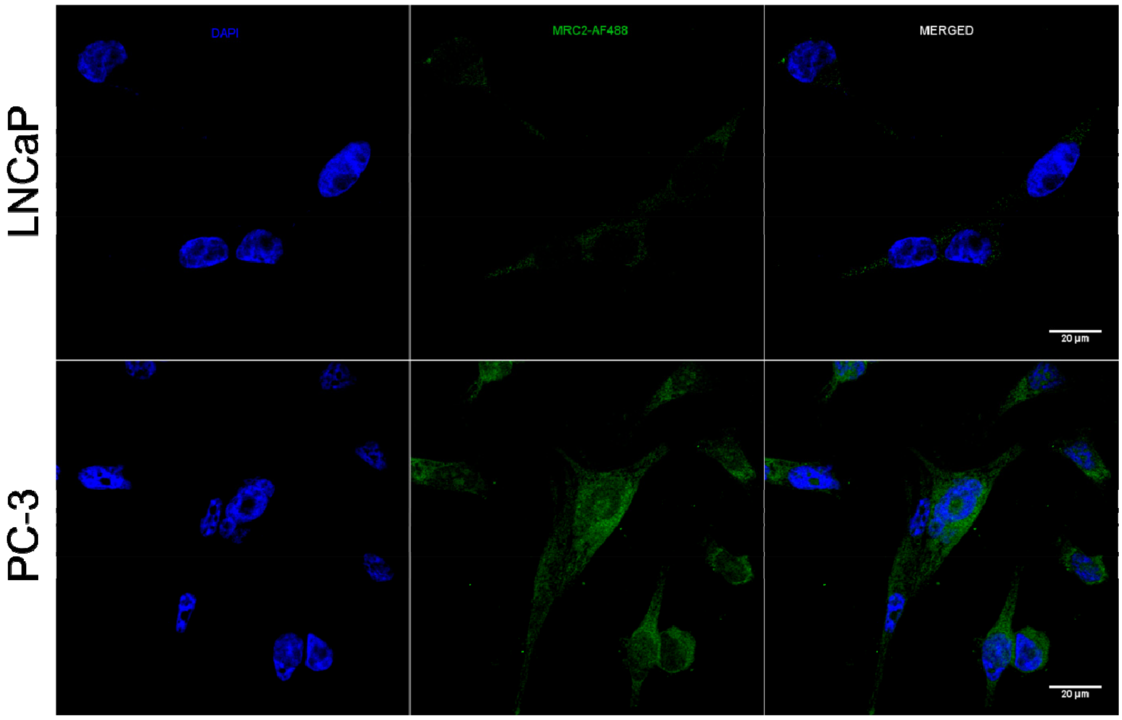

Supplementary Figure S7. LNCaP and PC-3 cells were analyzed by confocal laser microscopy imaging. Representative individual fluorescence images and merged images MRC2-AF488 (green) and nuclei counterstained with DAPI (blue) are shown. Scale bar = 20 µm.

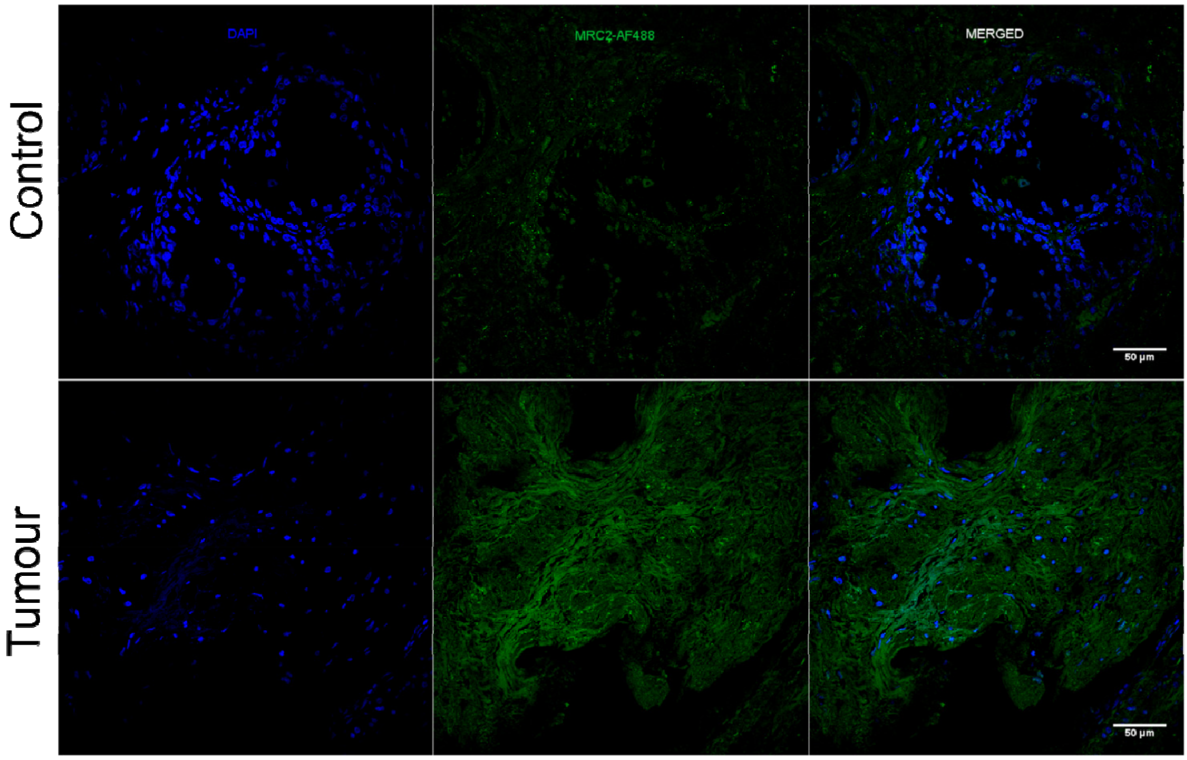

Supplementary Figure S8. Control and PC human prostate (ISUP 4) tissue sections were analyzed by confocal laser microscopy imaging. Representative individual fluorescence images and merged images MRC2-AF488 (green) and nuclei counterstained with DAPI (blue) are shown. Scale bar = 50 µm.

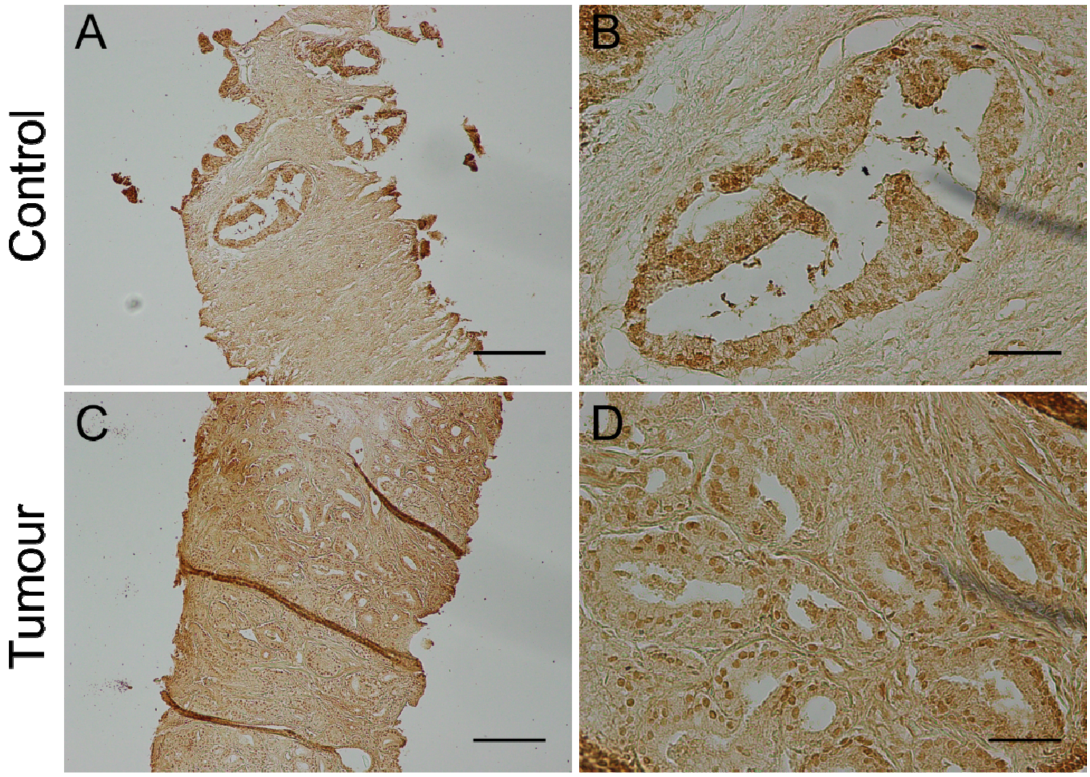

Supplementary Figure S9. Control and PC human prostate (ISUP 4) tissue sections were analyzed by light microscopy imaging. Primary antibody anti-MRC2, biotinylated secondary antibody and diaminobenzidine tetrahydrochloride (DAB) were used in immunodetection assay. Representative images of *MRC2* are shown. A and C scale bar = 200 μm; B and D scale bar = 50 μm.

BIBLIOGRAPHY

29. Safran, M.; Rosen, N.; Twik, M.; BarShir, R.; Stein, T.I.; Dahary, D.; Fishilevich, S.; Lancet, D. The GeneCards Suite. In *Practical Guide to Life Science Databases*; Springer Singapore: Singapore, 2021; pp. 27–56.

30. Salameh, A.; Lee, A.K.; Cardó-Vila, M.; Nunes, D.N.; Efstathiou, E.; Staquicini, F.I.; Dobroff, A.S.; Marchiò, S.; Navone, N.M.; Hosoya, H.; et al. PRUNE2 Is a Human Prostate Cancer Suppressor Regulated by the Intronic Long Noncoding RNA *PCA3*. *Proceedings of the National Academy of Sciences* **2015**, *112*, 8403–8408, doi:10.1073/pnas.1507882112.

31. Melander, M.C.; Jürgensen, H.J.; Madsen, D.H.; Engelholm, L.H.; Behrendt, N. The Collagen Receptor UPARAP/Endo180 in Tissue Degradation and Cancer (Review). *Int J Oncol* **2015**, *47*, 1177–1188, doi:10.3892/ijo.2015.3120.

32. Fei, F.; Qu, J.; Zhang, M.; Li, Y.; Zhang, S. S100A4 in Cancer Progression and Metastasis: A Systematic Review. *Oncotarget* **2017**, *8*, 73219–73239, doi:10.18632/oncotarget.18016.

33. Palmieri, C.; Caley, M.P.; Purshouse, K.; Fonseca, A.-V.; Rodriguez-Teja, M.; Kogianni, G.; Woodley, L.; Odendaal, J.; Elliott, K.; Waxman, J.; et al. Endo180 Modulation by Bisphosphonates and Diagnostic Accuracy in Metastatic Breast Cancer. *Br J Cancer* **2013**, *108*, 163–169, doi:10.1038/bjc.2012.540.
